# Modeling COVID-19 Transmission Dynamics With Self-Learning Population Behavioral Change

**DOI:** 10.3389/fpubh.2021.768852

**Published:** 2021-12-22

**Authors:** Tsz-Lik Chan, Hsiang-Yu Yuan, Wing-Cheong Lo

**Affiliations:** ^1^Department of Mathematics, City University of Hong Kong, Kowloon, Hong Kong SAR, China; ^2^Department of Biomedical Sciences, City University of Hong Kong, Kowloon, Hong Kong SAR, China

**Keywords:** COVID-19, mathematical modeling, population behavioral change, pandemic in Hong Kong, delay differential equation

## Abstract

Many regions observed recurrent outbreaks of COVID-19 cases after relaxing social distancing measures. It suggests that maintaining sufficient social distancing is important for limiting the spread of COVID-19. The change of population behavior responding to the social distancing measures becomes an important factor for the pandemic prediction. In this paper, we develop a SEAIR model for studying the dynamics of COVID-19 transmission with population behavioral change. In our model, the population is divided into several groups with their own social behavior in response to the delayed information about the number of the infected population. The transmission rate depends on the behavioral changes of all the population groups, forming a feedback loop to affect the COVID-19 dynamics. Based on the data of Hong Kong, our simulations demonstrate how the perceived cost after infection and the information delay affect the level and the time period of the COVID-19 waves.

## 1. Introduction

Coronavirus disease (COVID-19) is an infectious disease caused by a newly discovered coronavirus known as SARS-CoV-2 (1). According to the report of WHO in April 2021, over 132 million people were reported to be infected with COVID-19 and there were over 2.8 million deaths (2). As such, COVID-19 has been declared as a public health emergency of international concern on July 30, 2020. Since the beginning of the pandemic, many efforts were put into predicting the disease dynamics and suggesting optimal disease control strategies. Mathematical modeling is a major tool for COVID-19 prediction, for example using SIR (susceptible-infected-recovered) or SEIR (susceptible-exposed-infected-recovered) model to describe the dynamics of COVID-19 (3–5). Different variations of the SIR model were also studied, such as COVID-19 network models (6, 7) and a model with spatial impact on COVID-19 transmission (8).

Recurrent outbreaks of COVID-19 cases were observed in many locations. The existence of asymptomatic patients and the change of population behavior responding to the social distancing measures may be the factors for these recurrent outbreaks. Asymptomatic patients are individuals who are infectious but are not reported and show no symptoms. Because of the unawareness of infection status, asymptomatic infectious is not defined in SIR models. Recent research showed that the incubation period of COVID-19 could be as long as 12 days while the latent period is about 4 days (9). This result emphasizes the importance of the consideration of asymptomatic patients. For including the COVID-19 transmission with asymptomatic patients, some recent studies (10, 11) considered the extension of SIR model to SAIR model, incorporating a new compartment for asymptomatic patients.

**Figure 1:**
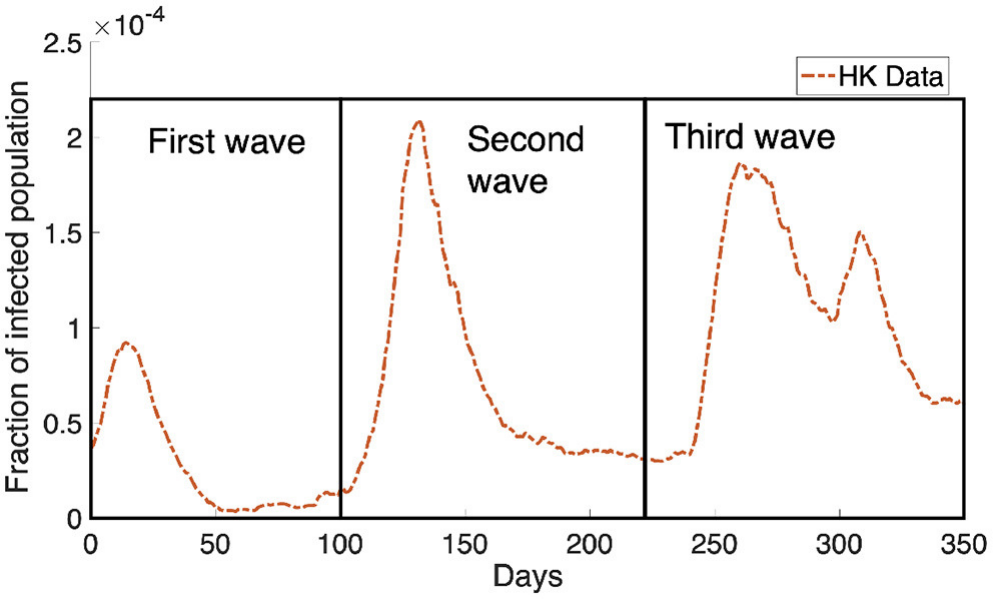
The Hong Kong infected population obtained from Department of Health, Government of HKSAR (20) is shown in the orange dotted curve. We can see the infected population rebound multiple times in the data, causing the dynamics of the infected population to look like waves. There are three waves where we have boxed them and marked them as the first wave, the second wave and the third wave as shown in the figure.

Another significant factor affecting COVID-19 transmission is related to the behavioral change of individuals. Policymakers enforced social distancing measures that aim to reduce the contact in a population. It is observed that 139 countries have implemented social distancing measures (12). Regulating social distancing has been shown to be an effective strategy in controlling the COVID-19 transmission (13–15) as it implies a behavioral change in population which will reduce the rate of COVID-19 transmission. In (16, 17), it was shown that individual behavior has a huge impact on the disease dynamics and sometimes leads to different predictions when comparing to a standard SIR model (18). Besides the intervention by the policymaker, individuals' decisions can be based on the perceived risk of infection (19) and the demands of a social environment (6). In general, the behavior change depends on the individual's utility which measures the balance between the risk of infection and the normal lifestyle. This adaptive behavior change may lead to the recurrent outbreaks of COVID-19 cases observed in many cities, such as Hong Kong (Figure 1).

The population decides the activity using the information about the infected population and the perceived cost after infection, including treatment fee and loss in economic productivity. How do the dynamics of COVID-19 transmission depend on the cost after infection and the time delay for receiving the information? In this paper, we will apply the data of Hong Kong to study how self-learning behavioral change affects the level and the time period of the COVID-19 waves. First, we will develop a SEAIR compartmental model for simulating the dynamics of COVID-19 transmission, in which the population can decide to reduce their activity outside or to have a normal lifestyle based on the evaluation of the utility functions. Then we will discuss the parameter estimation and perform numerical simulations to study the role of self-learning behavioral change in disease transmission.

## 2. Methods

In this section, we will develop a mathematical model for studying COVID-19 transmission. We first discuss an existing model from a recent study and then develop a novel model with a consideration of population behavioral change and asymptomatic patients.

Here we will define some terminologies and notations used in the paper. We separate the population into five compartments: susceptible compartment *S*, exposed compartment *E*, asymptomatic compartment *A*, infected compartment *I*, recovered compartment *R*. The susceptible compartment, the exposed compartment and the asymptomatic compartment are further divided into two types. We define the population who behaves normally as “normal activity type” and the population who reduces the frequency of outside activities as “reduced activity type.” In our model, the normal activity type is labeled by a superscript “^*n*^ ” and the reduced activity type is labeled by “^*r*^ .” For example, *S*^*n*^ represents the susceptible compartment that has normal activity and *A*^*r*^ represents the asymptomatic compartment that has reduced activity. We define that *S*^*n*^, *S*^*r*^, *E*^*n*^, *E*^*r*^, *A*^*r*^, *A*^*n*^, *I*, and *R* are the population numbers for the corresponding compartments and types, which are the functions of time *t*. The dot notation represents the derivative with respect to *t*, for example, $\overset{∙}{S^{n}}$ represents the derivative of *S*^*n*^ with respect to *t*.

### 2.1. Mathematical Model

In (19), based on the variables *S*, *I*, and *R* defined before, Amaral et al. studied the behavioral change in a SIR model:


(1) $$\left\{ \begin{array}{l} {\dot{S}}^{n}=-\beta_{N}I^{n}S^{n}-\beta_{a}I^{r}S^{n}+\rho \Phi_{S},\\ {\dot{S}}^{r}=-\beta_{N}I^{n}S^{r}-\beta_{Q}I^{r}S^{r}-\rho \Phi_{S},\\ {\dot{I}}^{n}=\beta_{N}I^{n}S^{n}+\beta_{a}I^{r}S^{n}+\rho \Phi_{I}-\gamma I^{n},\\ {\dot{I}}^{r}=\beta_{N}I^{n}S^{r}+\beta_{Q}I^{r}S^{r}-\rho \Phi_{I}-\gamma I^{r},\\ \dot{R}=\gamma (I^{n}+I^{r}), \end{array}\right.$$

where β_*a*_, β_*N*_, β_*Q*_ are the transmission rate, and γ is the recovery rate. The functions Φ_*S*_ and Φ_*I*_ are the rates that the population changes the behavior (normal activity or reduced activity), defined as


(2) $$\begin{array}{r} \Phi_{S}=S^{r}\left(S^{n}+I^{n}\right)\theta \left(p_{r},p_{n}\right)-S^{n}\left(S^{r}+I^{r}\right)\theta \left(p_{n},p_{r}\right), \end{array}$$

(3) $$\begin{array}{r} \Phi_{I}=I^{r}\left(S^{n}+I^{n}\right)\theta \left(p_{r},p_{n}\right)-I^{n}\left(S^{r}+I^{r}\right)\theta \left(p_{n},p_{r}\right), \end{array}$$

where *p*_*n*_ and *p*_*r*_ are the payoffs for the normal and reduced activity population, respectively. The payoffs depend on the perceived cost after infection and the infection probability under different types of activities. The function θ is the Fermi rule


(4) $$\begin{array}{r} \theta \left(p_{1},p_{2}\right)=\frac{1}{1+e^{-\left(p_{2}-p_{1}\right)/k}}, \end{array}$$

which gives the probability of population to change from the strategy with payoff *p*_1_ to the strategy with payoff *p*_2_. This rule is used in many literature (19, 21–24). The term $\frac{1}{k}$ gives the intensity of selection. Equations (2) and (3) depend on the probability (4) and the interaction rate among populations, which is based on the *social learning* in the behavioral game theory (25).

Based on (19), we develop a new mathematical model for capturing the spreading of COVID-19 with self-learning behavioral change. Here, we consider two more compartments: exposed patients *E* and asymptomatic patients *A*. So we will add four new variables, *E*^*r*^ and *E*^*n*^, *A*^*r*^ and *A*^*n*^, to system (1). Here the asymptomatic patients refer to some patients who are in their pre-symptomatic transmission period and will become infectious in next stage. In our model, the rate of the behavior change is based on the *self-learning* (25). This assumption is different from the model in (19) that is based on the *social learning*. We assume that, based on the information of the spreading of disease, each individual can unilaterally decide his/her own strategy which will affect the population distribution of the two activity types, normal activity type and reduced activity type. Other than these two types, we also consider that there are *M* different groups in the population. Each compartment that belongs to the *i*-th group is labeled by subscript “_*i*_.” For example, we can decide the groups according to the age groups of the population. The total number of the whole population is constant during the time interval [0, *T*] of the model where *T* denotes the final time of the simulation. The model considered in this paper is


(5) $$\left\{ \begin{array}{l} \dot{S_{i}^{r}}=a_{i}S_{i}^{n}-b_{i}S_{i}^{r}-\beta_{i}^{r}S_{i}^{r},\\ \dot{S_{i}^{n}}=-a_{i}S_{i}^{n}+b_{i}S_{i}^{r}-\beta_{i}^{n}S_{i}^{n},\\ \dot{E_{i}^{r}}=a_{i}E_{i}^{n}-b_{i}E_{i}^{r}-(1-\mu )\sigma E_{i}^{r}-\mu \sigma E_{i}^{r}+\beta_{i}^{r}S_{i}^{r},\\ \dot{E_{i}^{n}}=-a_{i}E_{i}^{n}+b_{i}E_{i}^{r}-(1-\mu )\sigma E_{i}^{n}-\mu \sigma E_{i}^{n}+\beta_{i}^{n}S_{i}^{n},\\ \dot{A_{i}^{r}}=a_{i}A_{i}^{n}-b_{i}A_{i}^{r}+(1-\mu )\sigma E_{i}^{r}-\lambda A_{i}^{r},\\ \dot{A_{i}^{n}}=-a_{i}A_{i}^{n}+b_{i}A_{i}^{r}+(1-\mu )\sigma E_{i}^{n}-\lambda A_{i}^{n},\\ \dot{I_{i}}=\lambda (A_{i}^{r}+A_{i}^{n})+\mu \sigma E_{i}^{r}+\mu \sigma E_{i}^{n}-\gamma_{i}I_{i},\\ \dot{R}=\sum_{i=1}^{M}\gamma_{i}I_{i}. \end{array}\right.$$

**Figure 2:**
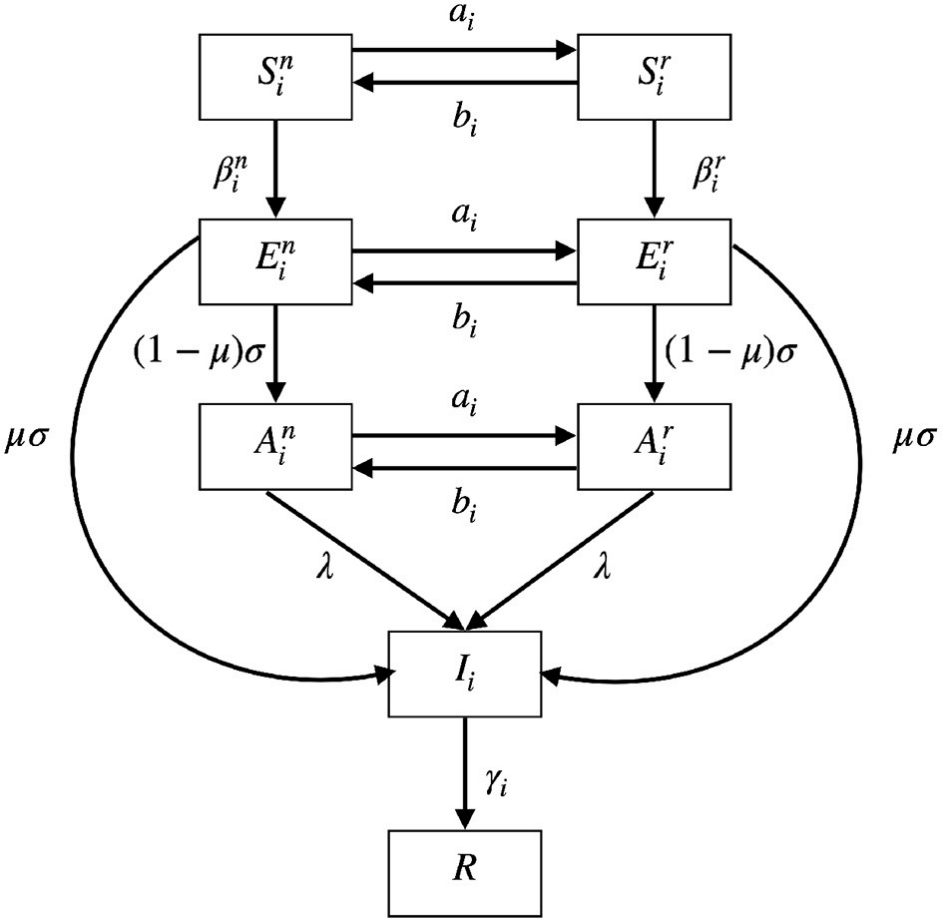
A schematic diagram of Model (5). *S* represents susceptible compartment; *E* represents exposed compartment; *A* represents asymptomatic compartment; *I* represents infected compartment; *R* represents recovered compartment. The populations can interchange between normal activity type ($S_{i}^{n}$, $E_{i}^{n}$, and $A_{i}^{n}$) and the reduced activity type ($S_{i}^{r}$, $E_{i}^{r}$, and $A_{i}^{r}$). The biological meaning of the parameters can be found in Table 1.

A schematic diagram of our model is given in Figure 2. The meanings of the functions and parameters used in Model (5) are listed in Table 1.

**Table 1:** Biological meaning of the function and the parameters used in Model (5).

**Variables**	**Biological meanings**
$S_{i}^{r}$	The *i*-th susceptible compartment with reduced activity
$E_{i}^{n}$	The *i*-th exposed compartment with normal activity
$E_{i}^{r}$	The *i*-th exposed compartment with reduced activity
$A_{i}^{n}$	The *i*-th asymptomatic compartment with normal activity
$A_{i}^{r}$	The *i*-th asymptomatic compartment with reduced activity
*I*_*i*_	The *i*-th infected compartment
*R*	The recovered compartment
**Parameters**	**Biological meanings**
*a*_*i*_	Rates at which $S_{i}^{n}$, $E_{i}^{n}$, and $A_{i}^{n}$ goes to $S_{i}^{r}$, $E_{i}^{r}$, and $A_{i}^{r}$.
*b*_*i*_	Rates at which $S_{i}^{r}$, $E_{i}^{r}$, and $A_{i}^{r}$ goes to $S_{i}^{n}$, $E_{i}^{n}$, and $A_{i}^{n}$.
μ	Probability that individuals show symptoms after exposed.
λ	Report rate of asymptomatic populations.
γ	Recovery rate of infected population.
σ	Rate at which exposed population become infectious.

The populations can interchange between normal activity type ($S_{i}^{n}$, $E_{i}^{n}$, and $A_{i}^{n}$) and the reduced activity type ($S_{i}^{r}$, $E_{i}^{r}$, and $A_{i}^{r}$) with rates *a*_*i*_ and *b*_*i*_. The two rates *a*_*i*_ and *b*_*i*_ will be defined in the later subsection. Susceptible populations $S_{i}^{n}$ and $S_{i}^{r}$ are infected at rates $\beta_{i}^{n}$ and $\beta_{i}^{r}$, respectively. The two rates $\beta_{i}^{n}$ and $\beta_{i}^{r}$ depend on the numbers of infected populations and will be explained later. After infection, the susceptible populations will become corresponding exposed populations $E_{i}^{r}$ or $E_{i}^{n}$. After the latent period $\frac{1}{\sigma }$ days, the exposed populations will show symptoms with probability μ and enter the infected population. Otherwise, if they show no symptoms with probability 1−μ, they will enter the asymptomatic populations. When the asymptomatic populations are reported through testing, contact tracing or developing symptoms after some time, they will become infected population. The report rate is denoted by λ. The infected population *I*_*i*_ is recovered at rate γ_*i*_ to the recovered population *R* which is immune to further infection as the reinfection rate is not high during the time period we considered.

### 2.2. Rate of Behavioral Change

Here we will introduce how the population decides to behave normally or reduce the frequency of their outside activities. There are two types of population, normal activity type and reduced activity type, in the *S*, *E*, and *A* compartments. The populations choose to alter their types, based on a self-learning process which depends on the utility functions.

The utility function of the individual in the *i*-th normal activity type at time *t* is defined as $v_{i}^{n}\left(z^{n},e\left(t\right)\right)$ which depends on the response function *z*^*n*^ and the environment vector *e* = [*I*_1_(*t*−τ), ⋯ , *I*_*M*_(*t*−τ)], where τ is the information delay. The individuals will select the optimal response function $z_{i}^{n*}:\left[0,T\right]\to \left[0,1\right]$ to maximize their own utility:


(6) $$\begin{array}{r} u_{i}^{n}\left(t\right):=\underset{z_{i}^{n}}{\text{max}}\;v_{i}^{n}\left(z_{i}^{n},e\left(t\right)\right), \end{array}$$

where $z_{i}^{n*}\left(t\right)=\underset{z_{i}^{n}}{\text{argmax}}\;v_{i}^{n}\left(z_{i}^{n},e\left(t\right)\right).$

The instantaneous optimization problem was applied in the discrete SIR model of the study (18). Here we consider it in the continuous model (5). Similarly we can define the utility for the reduced activity type. The individuals in the reduced activity type will select the optimal response function $z_{i}^{r*}:\left[0,T\right]\to \left[0,r_{\text{max}}\right]$ to maximize their own utility:


(7) $$\begin{array}{r} u_{i}^{r}\left(t\right):=\underset{z^{r}}{\text{max}}\;v_{i}^{r}\left(z^{r},e\left(t\right)\right), \end{array}$$

where $z_{i}^{r*}=\underset{z_{i}^{r}}{\text{argmax}}\;v_{i}^{r}\left(z_{i}^{r},e\left(t\right)\right)$. The feasible set for the reduced activity type is bounded above by a constant 0 < *r*_max_ <1 to reflect the reduced activity.

For the whole time interval [0, *T*], the function *z*^n*^:[0, *T*] → [0, 1] which represents the optimal activity outside for $S_{i}^{n}$, $E_{i}^{n}$, and $A_{i}^{n}$; $z^{r*}:\left[0,T\right]\to \left[0,r_{\text{max}}\right]$ which represents the optimal activity outside for $S_{i}^{r}$, $E_{i}^{r}$ and $A_{i}^{r}$.

The normal activity populations can choose to reduce their activity and enter the reduced activity populations at rate *a*_*i*_. Similarly the reduced activity populations can choose to become the normal activity populations at rate *b*_*i*_. The rates *a*_*i*_ and *b*_*i*_ are given as


(8) $$\begin{array}{r} a_{i}=\omega_{1}\theta \left(u_{i}^{n}\left(t\right),u_{i}^{r}\left(t\right)\right), \end{array}$$

(9) $$\begin{array}{r} b_{i}=\omega_{1}\theta \left(u_{i}^{r}\left(t\right),u_{i}^{n}\left(t\right)\right), \end{array}$$

where ω_1_ is a positive constant and θ is the Fermi Rule defined before. Here, *a*_*i*_ and *b*_*i*_ depend only on the utility function $u_{i}^{n}$ and $u_{i}^{r}$. They are different from the rates in Model (1).

### 2.3. Transmission Rate

In this subsection, we will discuss the transmission rates $\beta_{i}^{r}$ and $\beta_{i}^{n}$ in Model (5). Since there are two different strategies in the model, the population either can be a normal activity type or reduce the frequency of their activities. Thus we formulate two kinds of transmission rates, $\beta_{i}^{n}$ for the populations in the normal activity type, and $\beta_{i}^{r}$ for the populations in the reduced activity type. The transmission rates depend on the optimal function *z*^n*^ and *z*^r*^. Let *m*(*z*) represent the rate of contact made outside with the response function *z*. We assume that *m* is an increasing function of *z*.

Infection occurs when susceptible individuals make contact with the infectious compartment [$A_{i}^{n}$, $A_{i}^{r}$ and *I*_*i*_ in Model (5)] and that contact may lead to successful infection. The number of infection increases when the number of contact increases. From this assumption, we define that


(10) $$\begin{array}{r} \beta_{i}^{r}\left(z^{r*},t\right)=m\left(z^{r*}\right)\overset{M}{\underset{j=1}{\sum }}k_{n}A_{j}^{n}+k_{r}A_{j}^{r}+k_{I}I_{j}, \end{array}$$

(11) $$\begin{array}{r} \beta_{i}^{n}\left(z^{n*},t\right)=m\left(z^{n*}\right)\overset{M}{\underset{j=1}{\sum }}k_{n}A_{j}^{n}+k_{r}A_{j}^{r}+k_{I}I_{j}, \end{array}$$

where *k*_*n*_, *k*_*r*_ and *k*_*I*_ are the infection rates.

### 2.4. Value Functions

Now, we define the utility function in (6). The function $v_{i}^{n}$ models the internal decision process of behavioral change of individuals. It depends on the optimal value of *z*^n*^, the environment vector *e*(*t*−τ), and includes the present value and the expected cost in the future. This form is similar to the Bellman equation and has been used by other studies (18, 26, 27). Here, $v_{i}^{n}$ is defined as


(12) $$\begin{array}{r} v_{i}^{n}\left(t,e\right)=\underset{\text{Present value}}{\underset{︸}{\bar{v}\left(z_{i}^{n*}\left(t\right)\right)}}-\underset{\text{Expected cost after infection}}{\underset{︸}{c\omega_{2}m\left(z^{n*}\right)\overset{M}{\underset{j=1}{\sum }}k_{I}I_{j}\left(t-\tau \right)}}\\ \;-\underset{\text{Expected cost of susceptible}}{\underset{︸}{\left(1-\omega_{2}m\left(z^{n*}\right)\overset{M}{\underset{j=1}{\sum }}k_{I}I_{j}\left(t-\tau \right)\right)}}, \end{array}$$

where ω_2_ is a positive constant. In Equation (12), the present value is given by $\bar{v}\left(z_{i}^{n*}\left(t\right)\right)$. The perceived cost after infection is a product of the perceived cost *c* and the transmission rate without asymptomatic infection $m\left(z^{n*}\right)\overset{M}{\underset{j=1}{\sum }}k_{I}I_{j}\left(t-\tau \right)$. The transmission rate is different from Equation (11). Equation (12) does not depend on $A_{i}^{r}$ nor $A_{i}^{n}$ as we assume that individuals do not have any information about the asymptomatic population when making decision. We also assume that there is a time delay τ for the information. The delay τ is the time period that an individual becomes infected and this information reaches the decision-maker. The perceived cost after infection is related to treatment fee or loss in economic productivity but is not meant to be the exact measurement of the monetary value of the economical loss. It should be considered as the generic measurement and is relative to the cost of susceptible.

Similarly, we can define the value function for the reduced activity type as in (12) but *z*^n*^ is replaced by *z*^r*^:


(13) $$\begin{array}{l} v_{i}^{r}(t,e)=\underset{\text{Present value}}{\underset{︸}{\bar{v}(z_{i}^{r*}(t))}}-\underset{\text{Expected cost after infection}}{\underset{︸}{c\omega_{2}m(z^{r*})\sum_{j=1}^{M}k_{I}I_{j}(t-\tau )}}\\ \;-\underset{\text{Expected cost of susceptible}}{\underset{︸}{\left(1-\omega_{2}m(z^{r*}\sum_{j=1}^{M}k_{I}I_{j}(t-\tau )\right)}}. \end{array}$$

### 2.5. Parameter Estimation

First we provide the estimation of the parameters used in the numerical simulations. For the contact rate *m*(*x*), we set it as


(14) $$\begin{array}{r} m\left(x\right)=2.2x, \end{array}$$

which is based on (18). We define the present value $\bar{v}\left(x\right)$ as in (18),


(15) $$\begin{array}{r} \bar{v}\left(x\right)=x-\frac{x^{2}}{2}. \end{array}$$

The fraction of asymptomatic patient is based on the data from Department of Health, Government of HKSAR (20):


(16) $$\begin{array}{r} \frac{\text{number of reported asymptomatic patients}}{\text{number of reported infected patients}}=0.21. \end{array}$$

We assume that the probability of showing symptoms after infection is 0.79. We take $\lambda =\frac{1}{5.7\text{days}},\;\gamma =\frac{1}{10\text{days}},\;\sigma =\frac{1}{3\text{days}}$. Here, λ^−1^ is the mean time period of contact to illness onset, which is estimated from the study (28); γ^−1^ is the median recovery time of Remdesivir treatment, which is estimated from the study (29); the mean latent period, σ^−1^, is based on (30). We set *k* = 0.1 in the intensity of selection in the Fermi Rule as in (19). We summarize the parameter values mentioned above in Table 2.

**Table 2:** Parameters used in numerical simulations and the references.

**Parameters**	**Values**	**References**
μ	0.21	(20)
λ	$\frac{1}{5.7\text{days}}=0.1754{\text{days}}^{-1}$	(28)
γ	$\frac{1}{10\text{days}}=0.1{\text{days}}^{-1}$	(29)
σ	$\frac{1}{3\text{days}}=0.3333{\text{days}}^{-1}$	(30)
*k*	0.1	(19)

The reduced activity type is assumed to have a maximum activity at *r*_max_ = 0.8. The impact of other *r*_max_ will be studied in section 3. We assumed that *k*_*n*_ = 2*k*_*r*_ = 2*k*_*I*_. The cost after infection is assumed to be *c* = 10^6^ and the impact of varying the value of *c* will be discussed in the next section. The constants ω_1_ and ω_2_ are both set to be 1 in the simulations.

The initial condition used for the case of Hong Kong is based on the data from The Department of Health, Government of HKSAR (20). The simulation starts on March 25, 2020 which is the day that HK Government announced that border closures measure and all returning residents are subjected to Compulsory Quarantine Order (31). We consider all infected cases after this day are local cases. The final time for the simulation is *T*= 350 days. We assumed that initially the normal activity and reduced activity populations are both halves of the susceptible, asymptomatic and infected population. In the case of the simulations with more than one group, the population is distributed equally among the groups.

## 3. Results

For the long-term dynamics, the infected population will go to zero and the outcome will reach the disease-free equilibrium at the end. The intermediate dynamics of Model (5) is not trivial and we will apply numerical simulations to investigate the intermediate dynamics of Model (5). The detailed numerical scheme is discussed in Supporting Information.

### 3.1. Impact of Key Parameters

Based on the parameter setting before, we study the impact of some key parameters including the perceived cost after infection *c*, the time delay constant τ and the upper bound of reduced activity *r*_max_.

#### 3.1.1. Perceived Cost After Infection *c*

**Figure 3:**
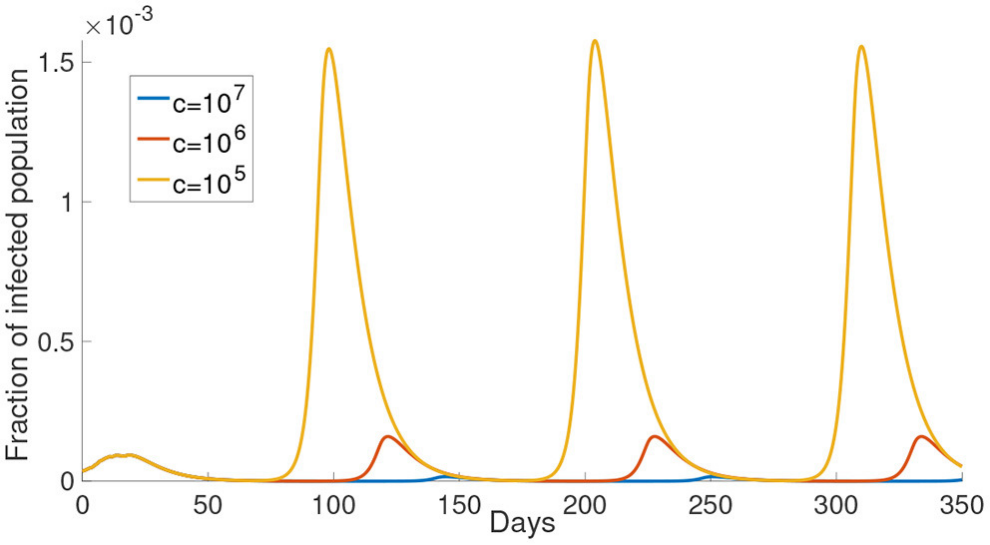
The effect of varying the cost after infection. The cost after infection is the scalar *c* in Equations (12) and (13). The infected population with different cost after infection is shown in different color. The corresponding *c* is shown in the legend.

In this subsection, we investigate the impact of varying the cost after infection. We consider the case that *M* = 1, that is, for example, the susceptible population is divided into $S_{1}^{r}$ and $S_{1}^{n}$. The perceived cost after infection, *c*, in Equations (12) and (13), is the perceived cost that the individuals need to pay if they are infected, which is related to the treatment fee or the loss in economic productivity due to sick leave. In Figure 3, we can see the simulations of the infected population with different values of *c*. As shown in Figure 3, the infected population is the least when *c* is the largest (*c* = 10^7^, yellow curve). When *c* decreases, the infected population increases. When the perceived cost *c* is small, such population may not choose to reduce their activity to prevent infection. In Equations (12) and (13), the second part is the expected cost, which depends on the perceived cost *c*. Thus, the lower the perceived cost after infection, the more the population will increase their activity to gain the optimal utility. Ultimately, this kind of response will contribute to more infections.

#### 3.1.2. Time Delay τ

**Figure 4:**
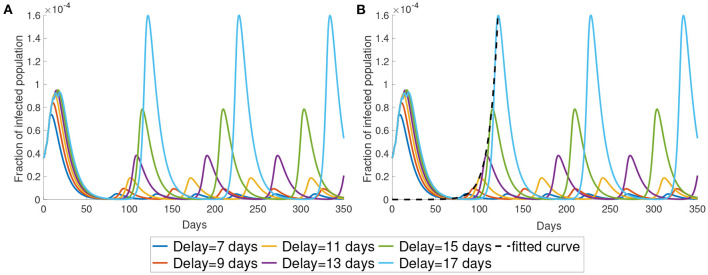
The impact of time delay. **(A)** We present the simulation with different delays. **(B)** We use exponential curve to fit the maximum in the first wave of the simulation. The fitted exponential curves are the black dotted line.

In this subsection, we study the dynamics of Model (5) under different values of time delay τ. The parameter τ represents the time lag between infected time and the moment that the information of the infected individuals reaches the decision-maker. This time delay is the sum of the latent period, the time period for testing and the delay of reporting. As shown in Figure 4, the time period between the maxima increases as the time delay increases. Also, a longer time delay will lead to a larger outbreak. The maximum of the infected population for each wave increases exponentially as the time delay increases. In Figure 4B, we plot the maximum of the first waves using the exponential form *ae*^*b* × days^. The fitted curve has the formula 5.61 × 10^−12^*e*^0.1335*t*^.

#### 3.1.3. Upper Bound of Reduced Activity *r*_max_

**Figure 5:**
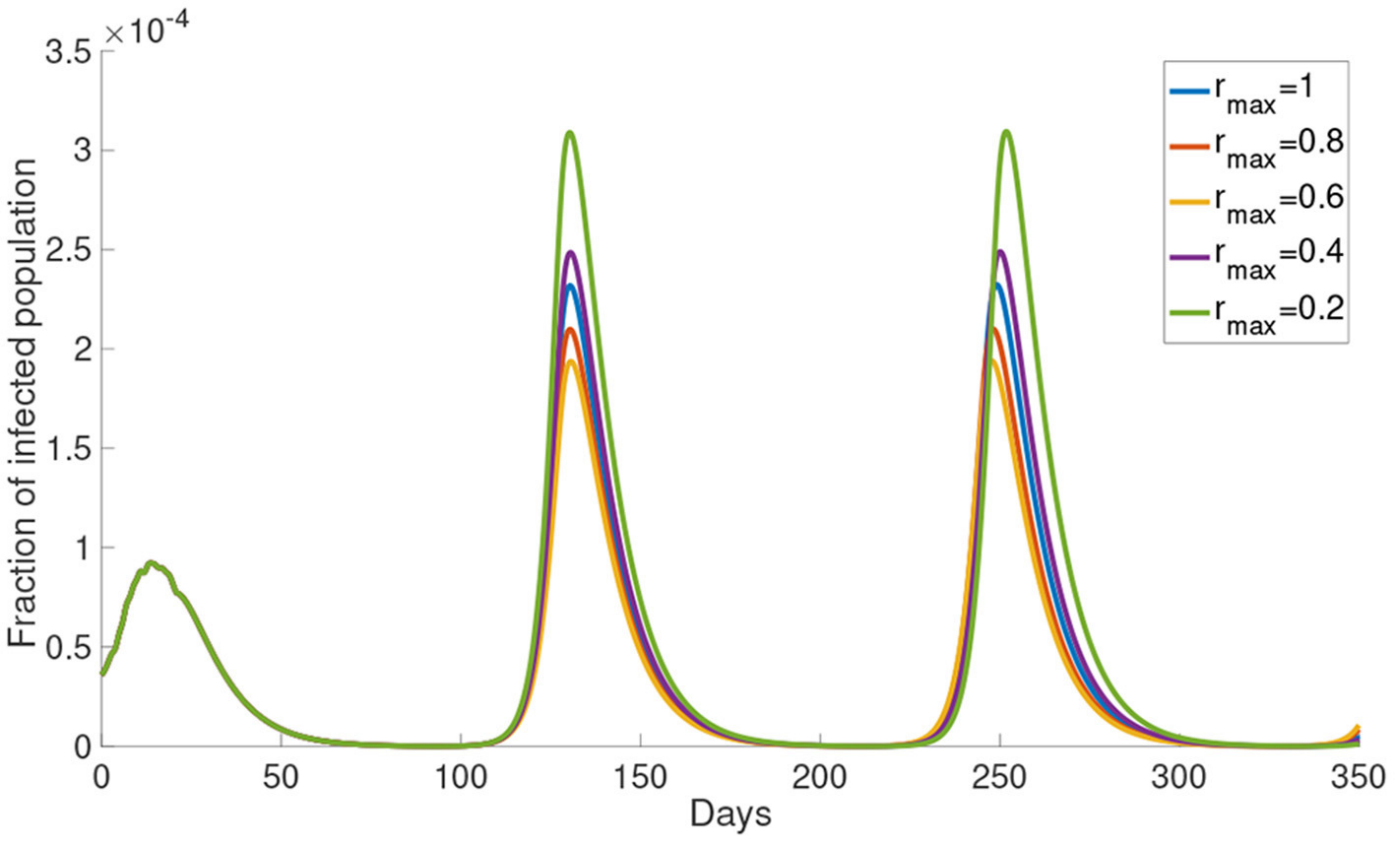
The simulation of the infected population with different *r*_max_.

Now, we study the dynamics of Model (5) with varying the upper bound of reduced activity *r*_max_. The simulations of the infected population with different *r*_max_ are shown in Figure 5. The infected population decreases as *r*_max_ decreases from 1 to 0.6. But the infected population increases as *r*_max_ decreases from 0.4 to 0.2. The local maximum in each wave of the infected population is the highest when *r*_max_ = 0.2 and the lowest when *r*_max_ = 0.6. This result shows that the infected population is not monotonically decreasing with *r*_max_. In the subsequent simulations we will use *r*_max_ = 0.8.

### 3.2. Simulation With the Data of Hong Kong

**Figure 6:**
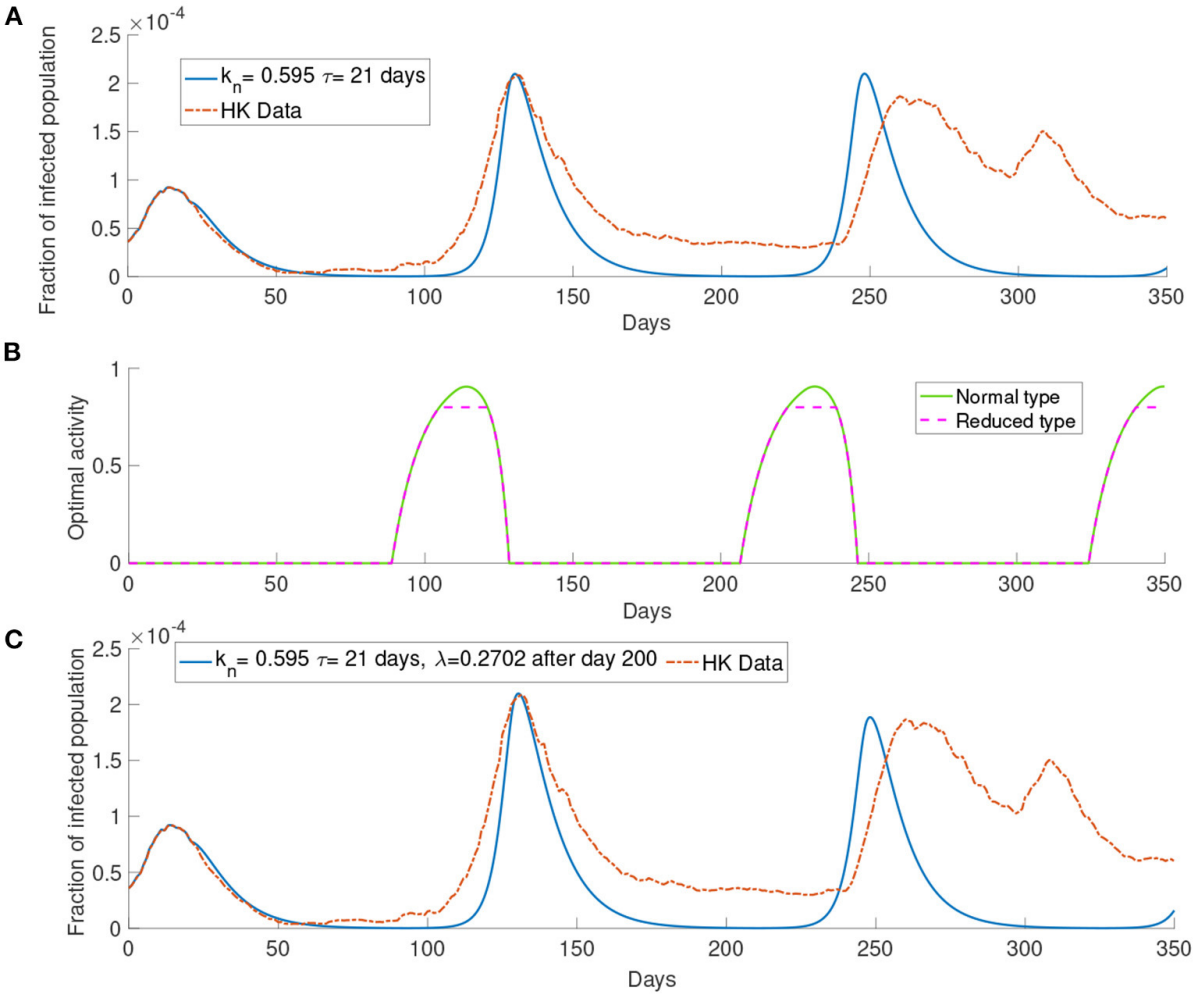
The simulation with the data of Hong Kong. **(A)** The comparison between the simulated and observed data. **(B)** The optimal activity. **(C)** The comparison of the infected population from the data and the simulation of our model with report rate λ increase to 0.2702 after day 200.

In this section, we present the simulation of our model comparing with the data of the infected population in Hong Kong. We first start with the case of *M* = 1. We set the parameters *k*_*n*_ = 0.595 and τ= 21 days. As seen in Figure 6, the simulation can produce several waves of infection. The number of infections reaches the local maximum point between day 100 and day 150 and decreases to a low level on around day 200. After day 200, the infected population starts to rise and reaches another local maximum on around day 250 before the infected population decreases to a low level again.

The infection waves are due to the change in the optimal activity as shown in Figure 6. As we can see in Figure 6 that when the infected population is at a low level, the optimal activity will increase and lead to a higher transmission rate; when the infected population is at a high level, the optimal activity will decrease and lead to a lower transmission rate. The third wave in Figure 6A, that is the wave between day 200 and day 250, is higher than the observed third wave in the data. As our simulation consider a long time horizon, many parameters may change along with time, such as the report rate λ. The Government in Hong Kong aims to improve the surveillance strategy by increasing the number of COVID-19 tests continuously (13). It would be reasonable to assume that the report rate will increase over time. In Figure 6C, we set the report rate λ to be $\frac{1}{3.7\text{days}}=0.2702{\text{days}}^{-1}$ after day 200 and observe a lower local maximum of the third wave which is similar to the one observed in the data.

### 3.3. The System With Bipartite Transmission Rates

In the previous simulation, we observe that the difference between the data and the simulation in the third wave of infection. To improve the accuracy of the simulation, we will investigate the situation where the transmission rate is modified to be the following form


(17) $$\begin{array}{r} \beta_{i}^{n}=\underset{\text{First part}}{\underset{︸}{m\left(z_{i}^{n*}\right)}}\left[k_{n}\underset{\text{Second part}}{\underset{︸}{m\left(z_{i}^{n*}\right)}}\overset{M}{\underset{j=1}{\sum }}A_{j}^{n}+k_{r}\underset{\text{Second part}}{\underset{︸}{m\left(z_{i}^{r*}\right)}}\overset{M}{\underset{j=1}{\sum }}A_{j}^{r}\right], \end{array}$$

(18) $$\begin{array}{r} \beta_{i}^{r}=\underset{\text{First part}}{\underset{︸}{m\left(z_{i}^{r*}\right)}}\left[k_{n}\underset{\text{Second part}}{\underset{︸}{m\left(z_{i}^{n*}\right)}}\overset{M}{\underset{j=1}{\sum }}A_{j}^{n}+k_{r}\underset{\text{Second part}}{\underset{︸}{m\left(z_{i}^{r*}\right)}}\overset{M}{\underset{j=1}{\sum }}A_{j}^{r}\right], \end{array}$$

where *k*_*n*_ = *k*_*r*_ is the infection rate. The biological meaning of this transmission rate is that the asymptomatic population has no symptoms and behaves like the susceptible population. For the normal activity type and the reduced activity type asymptomatic populations, similar to the susceptible population, we solve (6) and (7) to find out their optimal activity. The asymptomatic population contributes to less transmission if the population has a smaller optimal activity because a smaller optimal activity means less contact with other populations. The contact function in the first part models the contact made by the susceptible population. The contact function in the second part models the contact made by the asymptomatic population of the normal activity type and the reduced activity type. Thus, Equations (17) and (18) are bipartite transmission rates which involves the activity of the susceptible population and the asymptomatic population.

#### 3.3.1. Simulation With Single Group *M* = 1

**Figure 7:**
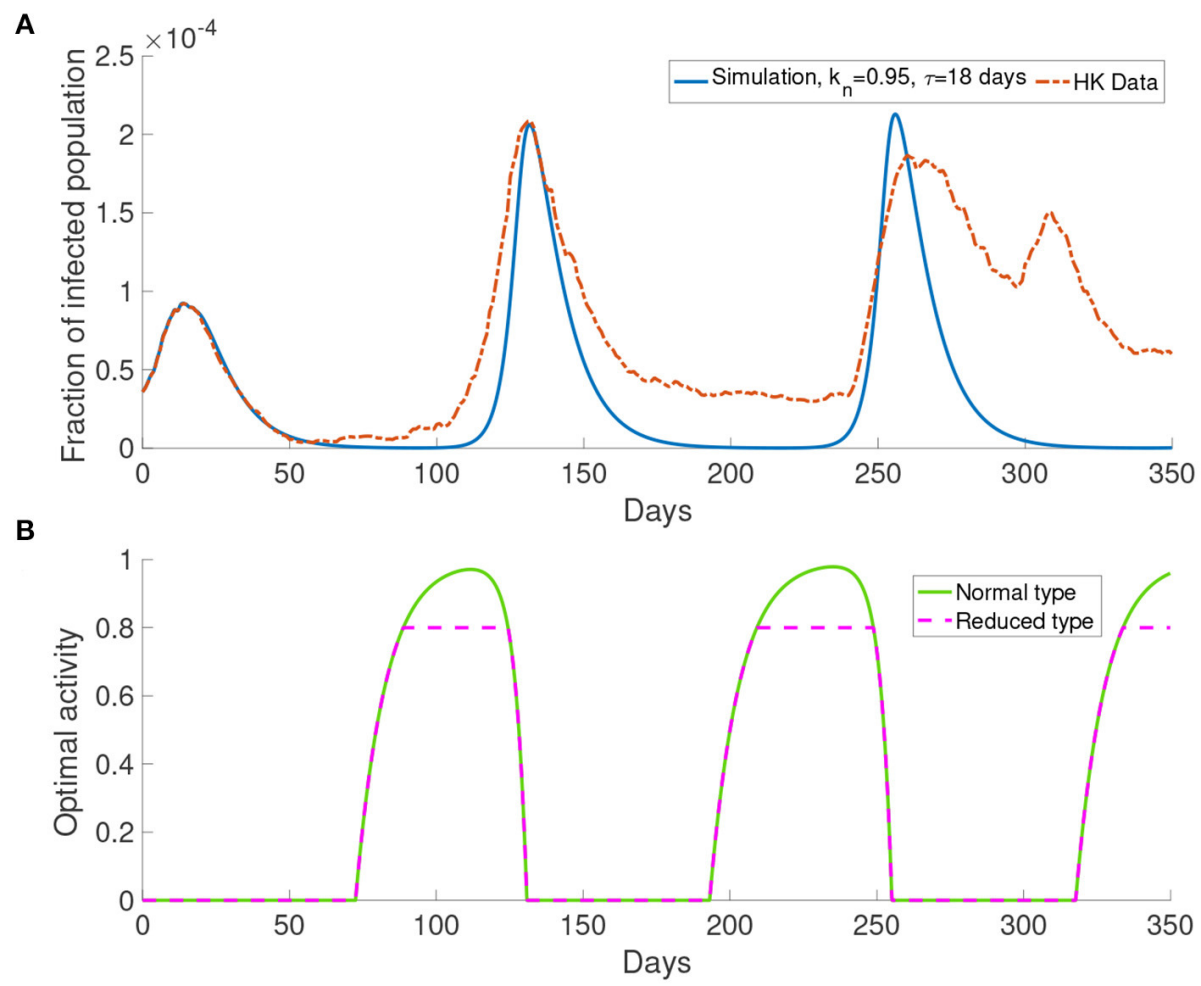
The simulation result with *M* = 1 and transmission rates in the form of Equations (17) and (18). **(A)** The comparison of data and the simulation. **(B)** The corresponding optimal activity for different types. Normal activity type and reduced activity type are the same except in the three intervals of behavioral change. They are from around day 75 to day 175, from around day 180 to day 250 and from around day 320 to day 350.

Here we present the simulation with *M* = 1 for the system with bipartite transmission rates. In the simulations, we set *k*_*n*_ = 0.95 and τ = 18 days. The cost after infection is 10^6^. The result shown in Figure 7 provides a better agreement with the third wave observed in the data.

Figure 7B shows the optimal activity for the normal activity type and the reduced activity type. We refer to the interval that shows different behavior for the normal activity type and the reduced activity type as *interval of behavioral change*. The optimal activity for the normal activity type and the reduced activity type is the same except in three intervals of behavioral change as depicted by Figure 7. The intervals of behavioral change are from day 75 to day 125, from day 180 to day 250 and from day 320 to day 350. On day 100, the population receives information about the infected population with 18 days delay, which corresponds to a moment with a small infected population (Figure 7A). On day 100, the normal activity type population perceives the risk of infection to be low and their optimal activity will be higher than the reduced activity type. Due to the definition of the reduced activity type, the reduced activity type will not increase their optimal activity despite having more utility. Finally, it causes the difference in the optimal activity in the intervals of behavioral change.

#### 3.3.2. Simulation With Multiple Groups *M*>1

Here we present the simulation when there are two groups, *M* = 2, for the population. We investigate the situation where the older people perceive the cost after infection to be higher than the young people as older people have longer recovery time (32) which will cost more in treatment fee. According to the discussion before, the behavioral change depends on the perceived cost after infection. Thus we define the first group corresponding to the older people who have higher perceived cost after infection *c* = 10^6^, and the second group corresponding to the middle-aged and young people who have lower perceived cost after infection *c* = 10^5^. We set the time delay τ = 15 days and *k*_*n*_ = 1.81. All other parameters are the same as those used before.

**Figure 8:**
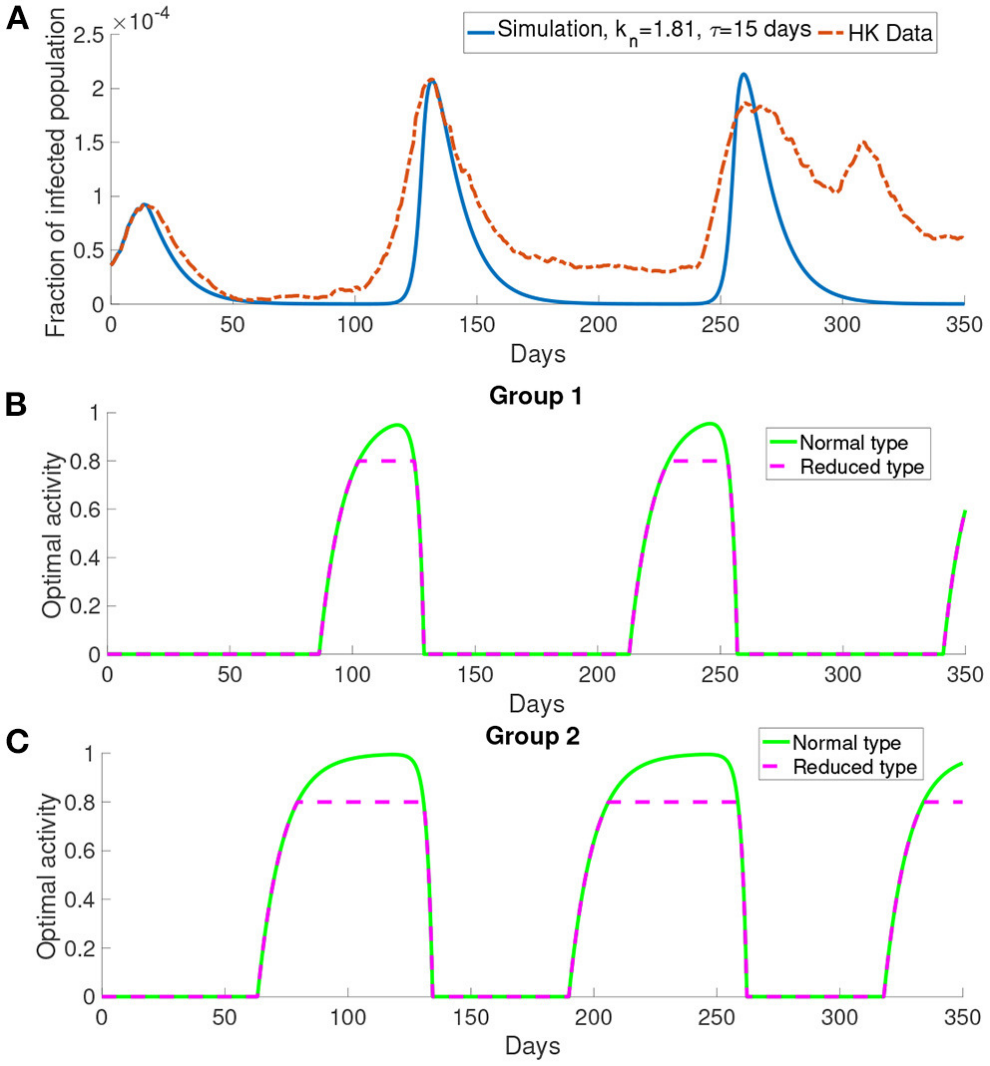
The simulation result with *M* = 2 and transmission rates in the form of Equations (18) and (17). **(A)** The comparison of the infected population in the simulation with the data. **(B,C)** The optimal activity of the two groups.

In Figure 8A, this simulation with two groups still produces wave-form dynamics of the infected population. By observing the susceptible population, the reduced activity type susceptible population decreases and the normal activity type susceptible population increases in the intervals of behavioral change. We can observe that there are three intervals of behavioral change shown in Figure 8. In these intervals of behavioral change, the utility will be larger for being the normal activity type. Figure 8 shows that the intervals of behavioral change are different for group 1 and group 2. Each interval of behavioral change of group 2 is longer than the interval of behavioral change of group 1. It means that the individuals in group 2, which have a smaller perceived cost after infection, increase their activity faster and remain their activity level longer than the individuals in group 1 which have a higher perceived cost after infection.

**Figure 9:**
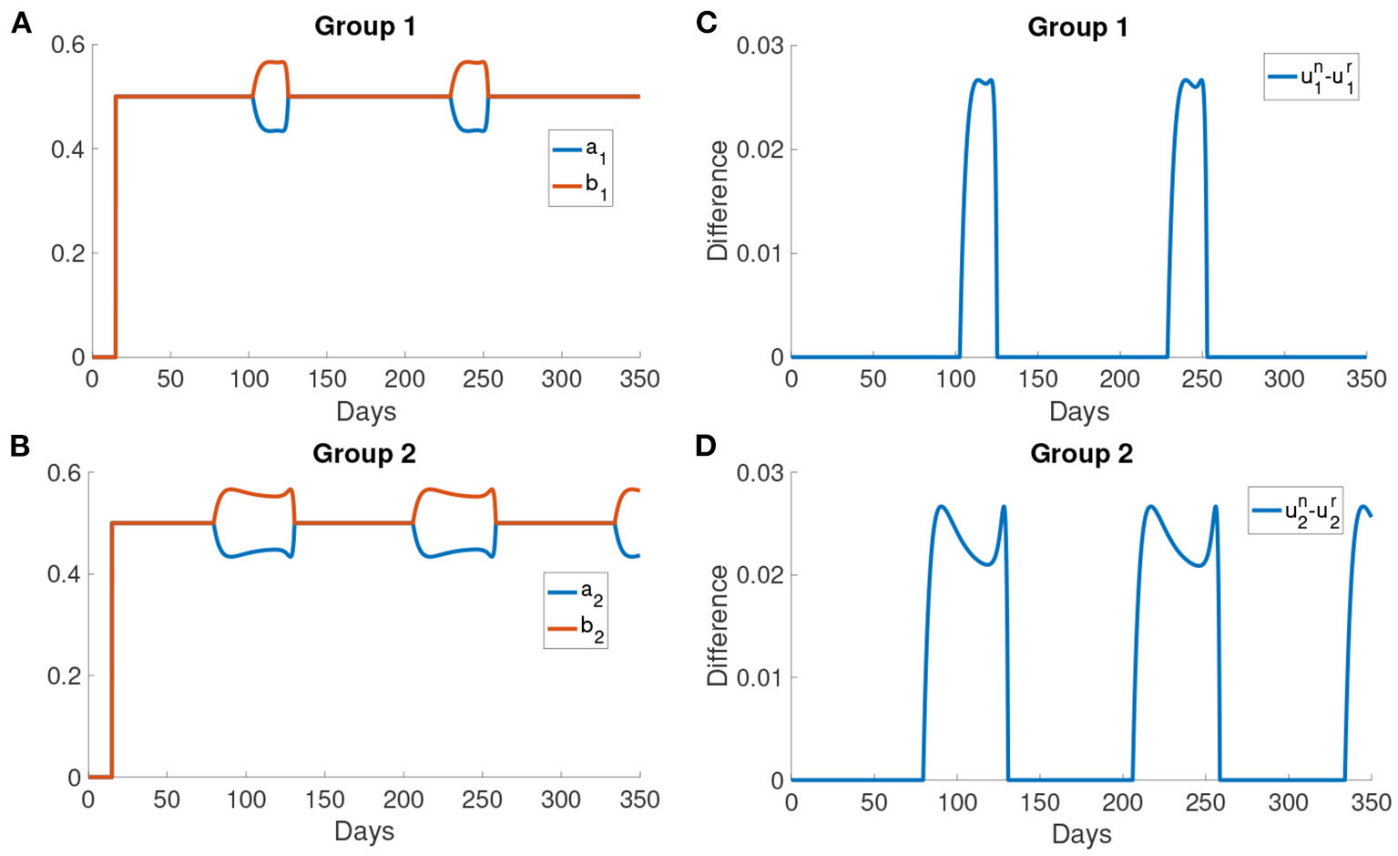
The difference in the utilities and the rates of change *a*_*i*_ and *b*_*i*_. **(A,B)** The difference in the utility of the normal activity type and the reduced activity type in different groups. The utility of normal activity type *u*^*n*^ and the reduced activity type *u*^*r*^ are calculated using Problem (6) and Problem (7). **(C,D)** The rates of changing type for different groups. *a*_*i*_ is the rate at which normal activity type becomes reduced activity type. *b*_*i*_ is the rate at which reduced activity type becomes normal activity type.

Figures 9A,B show the difference in the utility of the normal activity type and the reduced activity type. The utility of the normal activity type is always bigger than or equal to the reduced activity type since the activity of the reduced activity type is bounded by *r*_max_. Figures 9A,B show that the difference in the utility for group 2 forms a valley in each interval of behavioral change. The difference in the utilities controls the exchange rates *a*_*i*_ and *b*_*i*_. The two rates *a*_*i*_ and *b*_*i*_ are shown in Figures 9B,C. The normal activity type will gain more utility than the reduced activity type when the number of the infected population is small. As a result, the population in the reduced activity type will move to the normal activity type according to the Fermi Rule in the intervals of behavioral change.

After day 125, the activity of the normal activity type begins to decrease due to the delayed information of a large infected population has reached the normal activity type. Thus, the optimal responses of the two types are the same again as both of them are now below *r*_max_. *S*^*n*^ decreases to the initial level and then the normal activity type moves to the reduced activity type as the infected population becomes larger. *S*^*r*^ and *S*^*n*^ remain constant outside the intervals of behavioral change. Also, the rates *a*_*i*_, *b*_*i*_ and the utility are the same outside the intervals of behavioral change.

**Figure 10:**
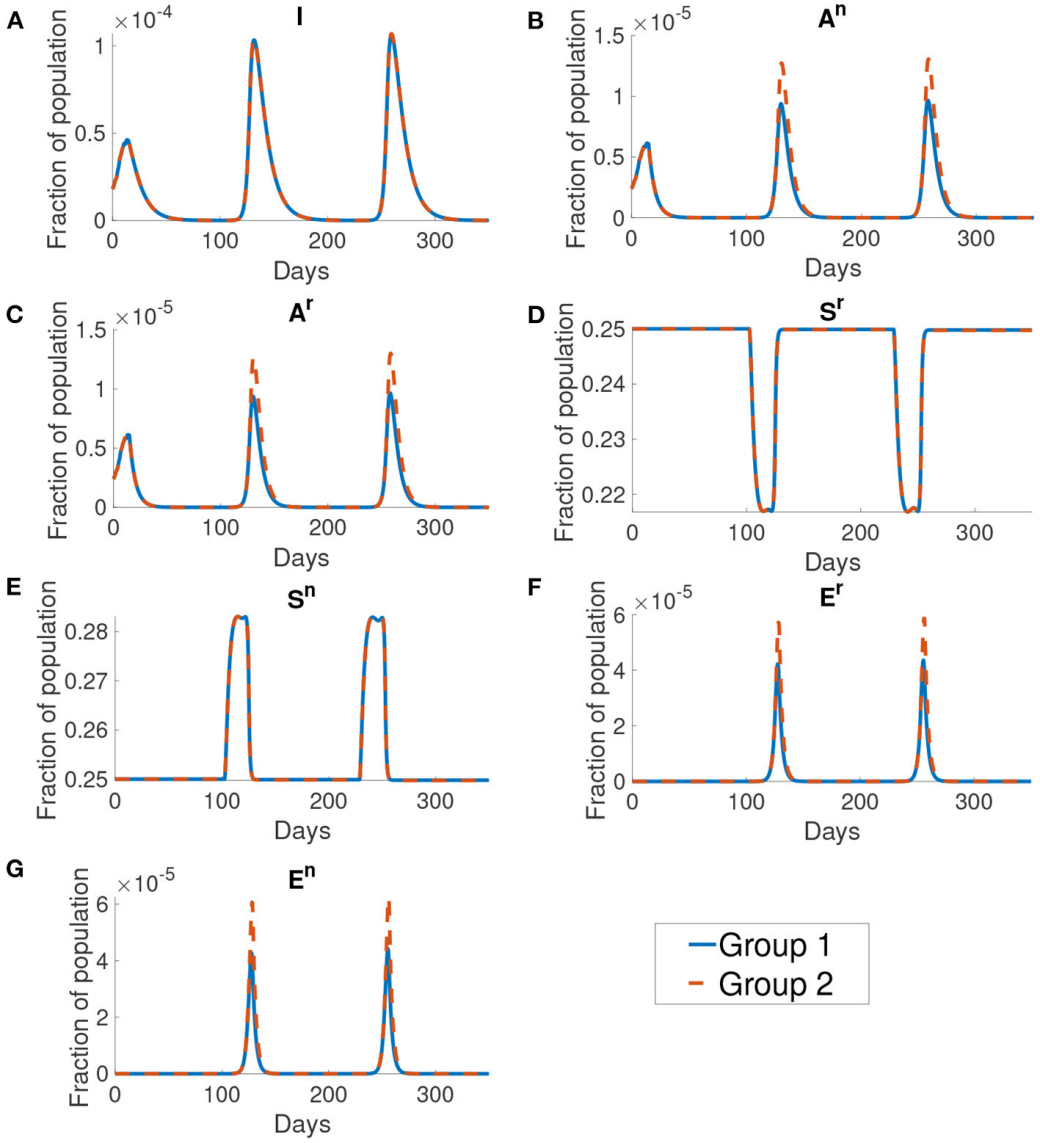
The dynamics of different compartments in the simulation. From figures **(A–G)** shows the infected population, the normal asymptomatic population, the reduced asymptomatic population, the reduced susceptible population, the normal susceptible population, the reduced exposed population and the normal exposed population. The blue curve shows the fraction of population for group 1 and the orange curve shows the fraction of population of group 2. We can see that the group 1 and group 2 are the same except at the normal asymptomatic and the normal exposed population.

Figure 10 shows the dynamics of different compartments. We can observe that the local maximum in each wave in the asymptomatic population (Figures 10B,C) constituted roughly 68% of all infected population. The asymptomatic population in group 1 is roughly 41% of the asymptomatic population in group 2 in both the normal activity type and the reduced activity type. But the infected population (Figure 10A) of group 1 and group 2 are the same.

**Figure 11:**
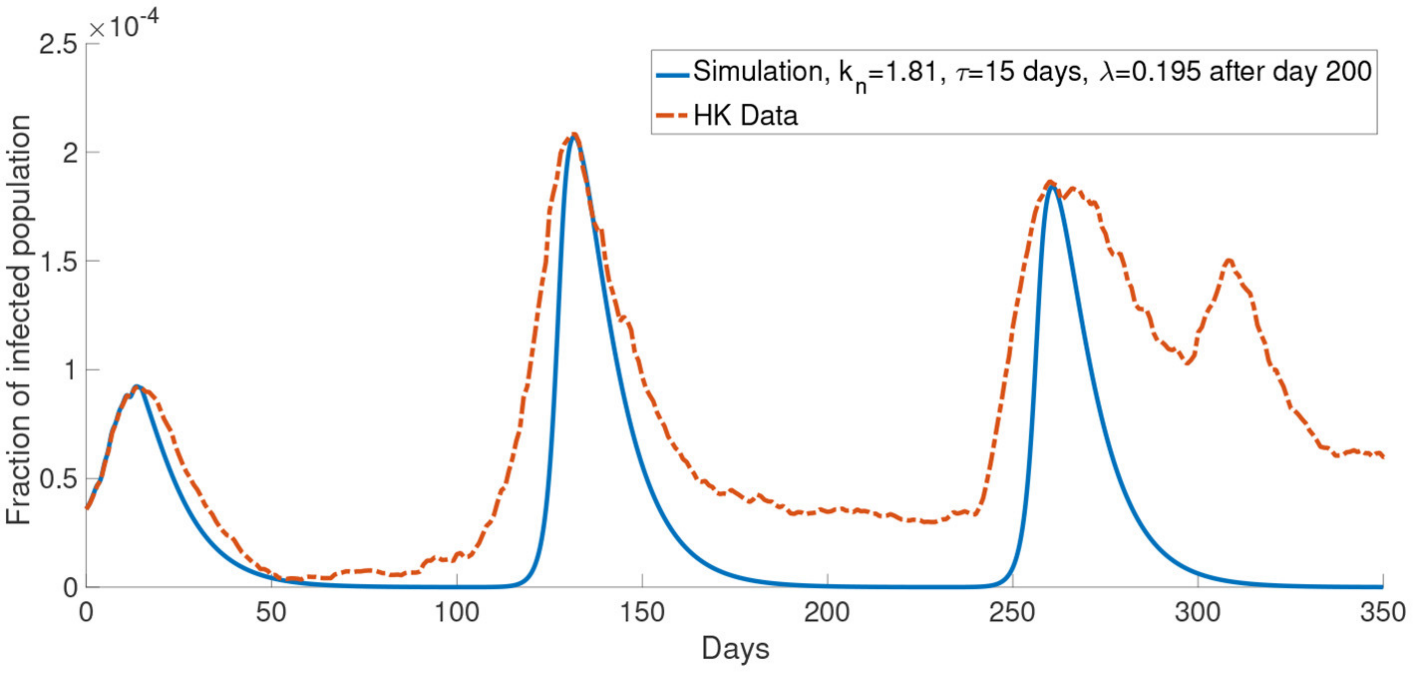
The comparison of the data and the simulation for the system with *M* = 2 with λ increased to 0.195 after day 200.

Similar to Figure 6A, Figure 11 shows a simulation with the report rate λ increased to 0.195days^−1^ after day 200. We observe a lower local maximum of the third wave which is similar to the one observed in the data.

Now, we consider more groups *M* = 3 for the population. We assume that the population is divided into three groups: young people, middle-aged people, and old people. In this case, we set the young people having *c* = 10^4^, the middle-aged people having *c* = 10^5^, the old people having *c* = 10^6^. This reflects a finer group division scheme for age-specific perceived cost after infection. We model the old people to have the highest cost after infection due to the highest treatment fee and longest recovery time (32), followed by the middle-aged people who have a medium cost after infection and the young people who have the lowest cost after infection since they often has a less economic burden and short recovery time. The cost after infection for group 1, group 2 and group 3 are 10^6^, 10^5^ and 10^4^, respectively. We set *k*_*n*_ = 0.865 and τ = 19 days. In our simulations, we observe that the properties of the system with more groups are similar to what we observed in the system with two groups. Figure 11 shows that the simulation with more groups can provide a better agreement with the real data.

## 4. Discussion

Many surveys suggest that different individuals in the population behave differently to COVID-19 due to various reasons, for example, age (33), gender (34, 35), political orientation (36–39), and education level (40). The aim of this study is to formulate a mathematical model for COVID-19 transmission with self-learning behavioral change in multiple population groups. In this paper, we have introduced a SEAIR compartmental model in which the transmission rates depend on the population's optimal activity. The population decides the optimal activity using the information about the infected population and the perceived cost after infection. We investigated the simulation with varying the cost after infection (*c*), the time delay for receiving the information (τ) and the upper bound for the optimal activity in the reduced activity type population (*r*_max_). The cost after infection is high in our model.

The model developed in our paper incorporated population behavior change which was discussed in some recent papers (18, 19, 41, 42). In (19), the authors consider the population dynamics as an evolutionary process. In their model, the population needs to learn from another population to make new decisions. One of the fundamental differences between our model and the model by Amaral et al. (19) is that we have relaxed their evolutionary assumption by allowing the population to change their strategy without contacting another population. Instead, the population changes their strategy based on what kind of information they received. We made this change in a way that is closely related to the daily life of people nowadays since people make their decision based on the information they receive from, for example, TV and newspapers. Nardin et al. (41) also studied the case where the population can change their strategy without meeting other populations. In their model, the rate of behavioral change is based on a discrete mechanism, unlike in our model where we used the Fermi rule for behavioral change. This also affects the dynamics of the infected population in which the waves of the infected population are less prominent in (41) than the waves in the models with a continuous rate of behavioral change in our study. In (42), the behavioral change with reduced activity is modeled as a percentage reduction in the transmission rate for the behavioral changed individual. This method works well for a relatively small time window of 5 months. In our study, the activity rate can vary with the utility function and be predicted in a longer time window of about 12 months.

We presented numerical simulations with unipartite transmission rates and bipartite transmission rates. Through the numerical simulations, we found that the mathematical model reproduces the observed waves of infection in Hong Kong. One main mechanism in our model that leads to the waves is the self-learning behavioral change. The population will choose an optimal response that balances the infection risk and the benefit from the outside activity. Our results suggest that the different waves of the infected population appear in the intervals of behavioral change. The interval of behavioral change is the time interval where the normal activity type population can obtain a higher utility by choosing a higher activity. A higher activity will lead to a higher transmission rate and more infections. Social distancing measures will alter the population behavior by lowering the population activity over a period of time, thus social distancing measure is an effective strategy in COVID-19 control. This supports the observed effectiveness of social distancing as a disease control measure (13–15).

In this paper, we investigated an alternate disease control measure that is in the absence of centralized agencies like the government. Instead, the disease control measure is initiated by individuals. In the model, the populations make a decision of reducing activity or not mainly based on the delayed information and their utility functions. Our simulations explained that different waves of the infected population are due to the individuals trying to balance the risk of infection and normal lifestyle. The perceived cost after infection and the delayed information are two determining aspects when individuals make a decision. As shown in Figure 3, for a smaller cost after infection, the infected population is larger. When the perceived cost *c* is small, the population will not reduce the chance of being infected by reducing the frequency of their activity. It will contribute to more infections. Another significant factor is the time delay τ. Long time-delayed information causes a larger outbreak. To effectively prevent an outbreak, we should decrease the time delay. This conclusion is consistent with the result in (43) which shows that the short time delay will reduce the number of the infected population. The numerical simulations suggest that information about the infected population should be disclosed as fast as possible to minimize the delay. Individuals with the latest information can make the best decision as a response to the disease. This implies that policymakers should let the population have full information about the consequence of infection.

**Figure 12:**
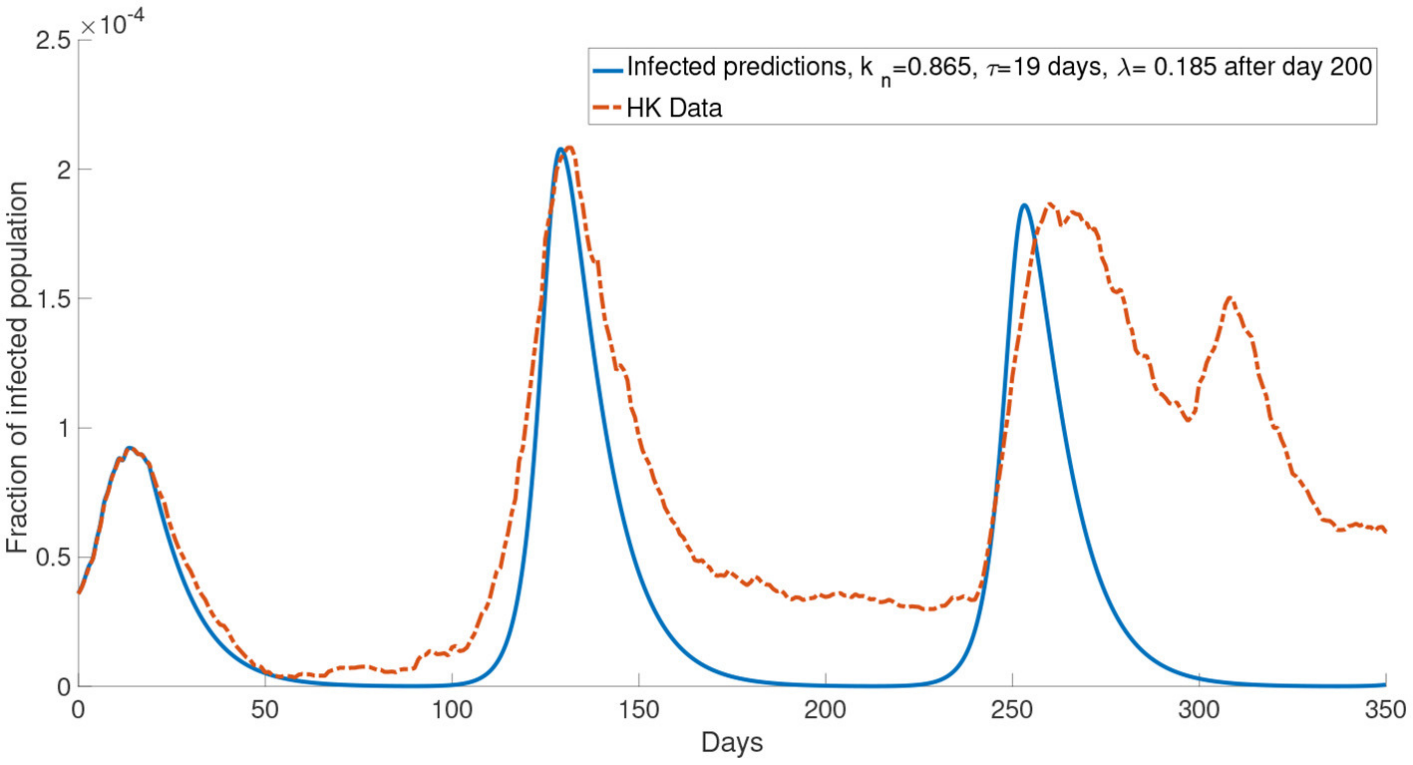
The comparison of the data and the simulation for the system with *M* = 3 with λ increased to 0.185 after day 200.

Although in our simulations, the local maximum in the second wave is similar to the data, the number of the infected population is less than the data in other times (for example, between day 100 and day 175 in Figure 6). One possible reason for this is that the mean-field assumption of a well-mixed population is not valid. It can be seen that our simulations reach the local maximum in a shorter time than the data from zero. This means that our simulations increase faster than the data. It is well-known that the infected population in a SIR model has exponential growth. Thus the data has a growth rate slower than exponential growth. It was shown that the population could be in the so-called small-world network, and this network leads to linear growth of the pandemic (44). We did not opt for a network model, but we used a continuous model because the network model is limited by computational power and speed, which is not feasible in simulating a large population. Apart from the underestimation of the infected population in some times, it can be seen that our model agrees quite well with the COVID-19 dynamics in Hong Kong for the first 150 days. But the numerical simulations appear to be inaccurate after 150 days. Specifically it seems that in most of our simulations (Figures 6–8), we overestimated the local maximum in the third wave. This inaccuracy is possibly due to our long prediction interval of 350 days. As the pandemic radically evolves, many of the parameters will change over these 350 days. For example, better disease control measures and increased usage of face masks could lower the transmission rate, a different variant of COVID-19 could increase the transmission rate and, better treatment methods could increase the recovery rate. In Figure 11, we investigated one such possible scenario in which the report rate increased over time. By introducing a time-dependent report rate, the numerical simulations, and the observed data showed a better agreement. The increase in report rate can lower the local maximum of the infected population as seen from the height in the third wave of the infected population in Figures 11, 12. This result suggests that increasing the report rate is a feasible COVID-19 control measure which is also noticed by the study in (45). One way to increase the report rate is by increasing the number of tests done, which was shown to be effective in practice (13, 46).

## Data Availability Statement

The original contributions presented in the study are included in the article/supplementary material, further inquiries can be directed to the corresponding author/s.

## Author Contributions

T-LC, H-YY, and W-CL: conceptualization and writing—review and editing. T-LC: formal analysis, methodology, and writing—original draft. T-LC and W-CL: investigation. W-CL: supervision. All authors contributed to the article and approved the submitted version.

## Funding

In this study, H-YY was supported by the Health and Medical Research Fund (COVID190215) and W-CL are partially supported by the CityU Strategic Research Grant (CityU 11301520).

## Conflict of Interest

The authors declare that the research was conducted in the absence of any commercial or financial relationships that could be construed as a potential conflict of interest.

## Publisher's Note

All claims expressed in this article are solely those of the authors and do not necessarily represent those of their affiliated organizations, or those of the publisher, the editors and the reviewers. Any product that may be evaluated in this article, or claim that may be made by its manufacturer, is not guaranteed or endorsed by the publisher.
